# Pragmatic randomised controlled trial of preferred intensity exercise in women living with depression

**DOI:** 10.1186/1471-2458-11-465

**Published:** 2011-06-12

**Authors:** Patrick Callaghan, Elizabeth Khalil, Ioannis Morres, Tim Carter

**Affiliations:** 1 Faculty of Medicine and Health Sciences, University of Nottingham, Queens Medical Centre, Nottingham, NG7 2HA, UK

## Abstract

**Background:**

Exercise may be effective in treating depression, but trials testing its effect in depressed women are rare.

**Aim:**

To compare the effect of exercise of preferred intensity with exercise of prescribed intensity in thirty-eight women living with depression.

**Methods:**

A Pragmatic RCT of 12 sessions of exercise at preferred intensity compared with 12 sessions at prescribed intensity. Beck Depression Inventory (BDI), Rosenberg Self Esteem Scale (RSES), General Health Questionnaire 12 (GHQ-12), heart rate (HR), Rating of Perceived Exertion Scale (RPE), Quality of Life in Depression Scale (QLDS), Multi-Dimensional Scale of Perceived Social Support (MDSPSS), SF12 Health Survey and exercise participation rates were compared between groups.

**Results:**

Intervention participants had statistically better BDI (*t *= 2.638, df = 36, *p *= 0.006, 95% mean (SD) 26.5 (10.7), CI-20.4 to -2.7, *d *= 0.86), GHQ-12 (*t *= 3.284, df = 36, *p *= 0.001, mean (SD) 8.3 (3.7) 95% CI -6.5 to -1.5, d = 1.08), RSES (*t *= 2.045, df = 36, *p *= 0.024, mean (SD) 11.3 (5.8), 95% CI 0.3 -6.4, d = 0.25), QLDS (*t *= 1.902, df = 36, *p *= 0.0325, mean (SD) 15.5 (7.9), 95% CI -12.2 -0.4, d = 0.27) RPE scores (*t *= 1.755, df = 36, *p *= 0.0475, mean (SD) 9.2 (3.2), 95% CI -.5 - 5.2, d = 0.77) and attended more exercise sessions (t = 1.781, df = 36, p = 0.0415, number of sessions 8 (65%), 95% CI-0.3 -4.8, d = 0.58). SF-12, MSPSS and HR did not differ significantly between groups.

**Conclusions:**

Exercise of preferred intensity improves psychological, physiological and social outcomes, and exercise participation rates in women living with depression.

**Trial Registration:**

ClinicalTrials.gov: NCT00546221

## Background

People with mental illness are more likely to suffer physical health problems and die prematurely than comparable populations who do not have mental illness, with recent evidence showing that being mentally ill increases a person's risk of ill-health and may shorten their life by 10 years [1]. Depression occurs in between 5% and 10% of people seeking primary care in the UK and is expected to be the second most common cause of disability worldwide; rates for women are double that of men [2]. Population studies report that depression is linked to low levels of exercise [3]. Exercise is reported as likely be beneficial in the treatment of depression [4-6] and recommended by NICE for the treatment of mild depression [7]. In line with previous reviews, a recent Cochrane systematic review of 25 depression trials found that exercise seemed to improve depressive symptoms [8], but there was little evidence of how effective it is, or the most effective type of exercise. Only six trials sampled clinical populations. When testing the effect of exercise on mental health outcomes among healthy people, researchers often use interventions based on national guidelines of intensity levels thought to produce health benefits [9]. Notwithstanding the benefits of exercise, national and international studies show that many people with mental health problems do not engage in physical activity, those that do, often do not maintain the prescribed intensity [10], as result, high attrition rates are reported [11]. Exercise that is matched to participants' preferred intensity improves mental health outcomes and improves attrition rates [12]. Our earlier work among young people suggests that tailored interventions supplemented with motivational support may increase self-esteem and overall quality of life, and reduce exercise attrition rates [13,14]. In this study we addressed the question: does exercise of preferred intensity lead to better psychological, physiological and social wellbeing outcomes and improved adherence rates when compared with exercise of prescribed intensity?

## Methods

### Design

We used a Pragmatic RCT (PRCT). The intervention group received twelve sessions of treadmill aerobic exercise of preferred intensity in groups of up to five, three times per week for four weeks. Preferred intensity, chosen exertion level, was established using the RPE scale [15]. The active comparator group received twelve sessions of treadmill aerobic exercise of prescribed intensity in groups of up to five, three times per week for four weeks. Prescribed intensity was exercise of an intensity and duration as recommended by national guidelines [10]. As the peak RPE scores in table 1 show, at baseline the intensity level of both groups was similar, this is what we anticipated. However, at the end of the study, the mean peak RPE score for the active comparator arm was significantly higher showing they were exercising at a higher level of intensity closer to prescribed levels, whilst the participants in the intervention arm were exercising closer to the preferred intensity levels which was the goal of the study. The exercise sessions were supervised by a qualified exercise therapist. Both the intervention and active comparator programmes received manualised psychosocial support through motivational interviewing and advice on maintaining healthy lifestyles around exercise from a qualified health psychologist. The manualised psychosocial support was based on the Transtheoretical Model of Change [12] and in particular the self-efficacy construct shown by the researchers [16] to be a strong predictor of exercise intentions and actual exercise behaviour.

**Table 1:** Primary and secondary outcomes by study arm¹

	Intervention arm	Active comparator arm (*n *= 19)	Mean difference (*n *= 19) (95% confidence interval)	*p *value
*Primary outcome*				
Mean BDI score (SD)				
Baseline	26.5 (10.7)	30.5 (12.0)		
Plenary	18.1 (13.0)	29.6 (13.9)		
Mean change	-8.5 (9.8)	-0.9 (6.6)	-11.5 (-20.4 to -2.7)	0.006
*Secondary outcomes*				
Mean GHQ score (SD)				
Baseline	8.3 (3.7)	8.8 (3.4)		
Plenary	3.5 (3.1)	7.5 (4.3)		
Mean change	-4.8 (5.3)	-1.3 (2.5)	-4.0 (-6.5 to -1.5)	0.001²
Mean SF-12 score (SD)				
Baseline	25.9 (6.2)	25.6 (7.5)		
Plenary	30.0 (8.7)	26.4 (7.2)		
Mean change	4.1 (7.3)	0.8 (2.8)	3.6 (-1.7 to 8.8)	0.08
Mean RSES score (SD)				
Baseline	11.3 (5.8)	11.0 (4.9)		
Plenary	14.7 (4.8)	11.5 (4.9)		
Mean change	3.4 (3.1)	0.5 (3.0)	3.2 (0.3 to 6.4)	0.024
Mean MSPSS score (SD)				
Baseline	47.6 (18.3)	51.6 (15.4)		
Plenary	52.1 (16.7)	58.1 (16.9)		
Mean change	4.5 (10.1)	6.4 (19.6)	5.9 (-5.1 to 17.0)	0.14
Mean QLDS score (SD)				
Baseline	15.5 (7.9)	20.3 (8.5)		
Plenary	12.3 (7.2)	18.2 (11.4)		
Mean change	-3.3 (3.1)	-2.1 (7.7)	-5.9 (-12.2 to 0.4)	0.0325
Mean Attendance (×/12)(SD)	8.2 (3.6)	5.9 (4.2)	2.3 (-3.14 to 4.8)	0.0415
*Physiological outcomes³*				
Mean heart rate @ time 0 (beats/min) (SD)				
Baseline	87.5 (15.8)	92.8 (5.6)		
Plenary	95.1 (18.5)	96.6(11.7)		
Mean change	4.4 (15.7)	2.0 (6.5)	1.5 (-12.2 to 15.8)	0.414
Mean peak heart rate (beats/min) (SD)				
Baseline	109.6 (21.1)	121.3 (15.8)		
Plenary	121.1 (18.2)	136.8 (29.5)		
Mean change	6.7 (17.2)	8.2 (17.5)	15.7 (-6.4 to 37.9)	0.77
Mean RPE score @ time 0 (SD)				
Baseline	9.2 (3.2)	8.6 (3.3)		
Plenary	7.9 (2.6)	9.4 (3.6)		
Mean change	-0.7 (2.0)	0.4 (1.1)	1.5 (-1.4 to 4.3)	0.144
Mean peak RPE score (SD)				
Baseline	10.4 (2.8)	10.5 (2.9)		
Plenary	9.8 (2.7)	12.2 (3.5)		
Mean change	-0.3 (1.9)	0.9 (1.7)	2.4 (-0.5 to 5.2)	0.0475

^1 ^Independent *t*-tests.

^2 ^Unequal variances.

^3 ^Sub-sample: Intervention arm *n *= 11,active comparator arm *n *= 10.

### Qualitative study

In addition to the pragmatic trial, we added a qualitative component to the study in the form of focus groups with participants from the intervention and active comparator arms of the study. The purpose of this component was to provide information on the processes that might help to explain the quantitative outcomes, a technique used and recommended by previous researchers conducting pragmatic trials [17]. For reasons of space, we do not report the results of the qualitative study here, but refer to it in the discussion where it helps to elucidate our findings.

### Sample

**Figure 1:**
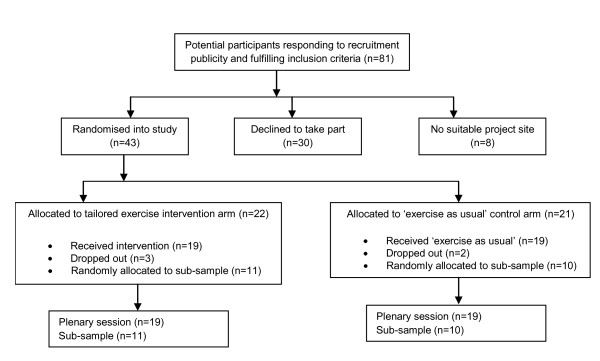
**Flow of participants through the study**.

Figure 1 shows the flow of participants through the study. Women were included if they were being monitored by, or receiving treatment for depression from any primary or secondary mental health service, aged 45-65 (age at first session of programme), living in the community and resident within Nottinghamshire as indicated by postcode. Participants were excluded if, at the time of the study, they were unable to participate on account of any injury or physical health problem. We estimated based on a predicted effect size on the primary outcome of around 0.82, at 80% power, with a 5% alpha level and predicted attrition of around 10%, a sample size of 58.

### Recruitment

The research team set up a project website, carefully piloted and targeted at the sample group, with an automated system for acquiring more information about the project. A dedicated telephone line with a named member of the project team, a female, was also provided. A poster/flyer was designed to circulate widely, again carefully targeted to appeal to the intended participant group. We sent information letters to General Practitioners (GP) and secondary services (local Mental Health Services), detailing the study and inviting clinicians to assist us in identifying potential participants. An assent form was included for each service/clinician to return if potential participants agreed to do this. Those services agreeing to engage with us received a pack containing a poster to display where potential participants could see it, a set of flyers to hand to potential participants, and a flow chart to guide each clinician through the inclusion criteria. Posters presented very clear information about eligibility criteria, to avoid any potential disappointment to interested parties. Clinicians were requested to hand the flyer out to every consecutive potential participant (to maximise opportunity for interested women to participate and minimise selection bias), having worked through the inclusion criteria. In the event of the potential participant wanting to ask further questions at this stage, the clinician was instructed to refer them to the project telephone hotline number provided on the flyer, where they could contact a named member of the project team. The clinicians' flow chart also included a reminder to the potential participant that this was a research study and not a course of prescribed treatment.

In addition, our collaborating service user group and the local hub of the national Mental Health Research Network (MHRN) publicised the programme among its members and relevant independent and voluntary sector organisations and contacts, using the project promotional poster and information flyer. MHRN staff also made visits to interested organizations using a lay-language information sheet of their own design.

### Procedure

This study was adopted by the MHRN local hub and was registered with the U.S. National Institutes of Health clinical trials database (https://register.clinicaltrials.gov). The study received a favourable ethical opinion from a local Research Ethics Committee and research governance approval from three participating healthcare Trusts.

Potential participants picked up an information flyer from a user group, saw the posters, or had a flyer handed to them by their clinician. The flyer/poster instructed them to visit the project website and/or to call a project telephone number if they were interested in participating. The researcher answering the phone screened the potential participant using the inclusion/exclusion criteria. Participants were invited to ask questions at this stage. Provided the potential participant indicated a continued desire to participate, their contact details were taken by the researcher, and a further information pack was sent out, describing the exercise programme in full with an acceptance slip to be completed and returned in a prepaid envelope. An important part of the pack was a reminder to participants to check with their GP prior to commencing the exercise programme if they had doubts about their fitness to participate. A standard letter to the participants' GP was included in the pack.

Provided they were eligible, upon receipt of the acceptance slip, the researcher forwarded the assigned participant code to an independent operative at the MHRN. The MHRN staff filled in the participant code on the next available programme slot which could be intervention or control, decided at random. Participants were allocated to the gym appropriate to their home address (to minimise inconvenience in travelling). Upon attendance at the introductory session of the programme, participants were talked through the information sheet and issues around random allocation. They were invited to ask questions, reminded of their right to withdraw at any stage, and formal written consent was obtained. This two-stage consent procedure with consent-in-principle, followed by formal face-to-face written consent helped the team manage the logistics of random allocation along with convening the exercise programmes in a spread of geographical locations. Reminder telephone calls were made to participants one week before their programme was due to commence, letters were sent in the absence of a telephone, timed to arrive one week before. Participants were once again encouraged to raise any questions and reminded to check with their GP prior to commencing the programme.

### Randomisation

Consenting participants were randomly allocated prior to inclusion into the intervention or active comparator by a research professional unconnected to the study, using a computer generated random sequence list from http://www.random.org. Following randomisation, three participants dropped out of the intervention arm, one person dropped out due to viral illness and two declined to continue, in the active comparator arm one reported the exercise as too taxing, and one declined to continue.

## Outcome Measures

### Beck Depression Inventory-II [18]

The BDI-II, the primary outcome measure, consists of 21 items to assess the likelihood and intensity of depression in clinical and non-clinical samples. Each item is a list of four statements arranged in increasing severity about a particular symptom of depression, scored on a scale from 0 to 3. The cut-offs are 0-13 - minimal depression; 14-19 - mild depression; 20-28 -moderate depression; and 29-63 - severe depression. Higher total scores indicate more severe depressive symptoms. We used the BDI as it is a measure of depression widely used in research where depression scores are a primary outcome measure and to allow us to better compare our results with previously published systematic reviews and trials.

### General Health Questionnaire -12[19]

The GHQ-12 screens for non-psychotic psychiatric disorders and is recommended for research purposes. This study used Goldberg's original scoring bimodial method. In this method response categories score 0, 0, 1, and 1 respectively. This gives scores ranging from 0 to 12. A score of 4 or more is considered indicative of 'caseness' - mental health problems.

### SF-12-II Health Survey [20]

The SF-12 is a self-reporting multipurpose scale used for assessing health-related quality of life for eight concepts of physical and mental health: physical functioning, role limitations due to physical health problems, role limitations due to emotional health problems, social functioning, emotional well-being, pain, energy and/or fatigue, and general health perceptions. SF-12 is a standardized measure of health status. Items are scored on Likert scales; a high score indicates poor health.

### The Rosenberg Self-Esteem Scale [21]

The RSES is a standardized scale and was used to assess global self-esteem and self-acceptance. This is a ten item self-rating Likert scale with items answered on a four point scale, from strongly agrees to strongly disagree. The higher the score, the higher the self esteem, 14-25 is considered normal, below 14 indicates low self-esteem.

### The Quality of Life in Depression Scale [22]

The QLDS is a standard measure of needs-based quality of life of patients with depression. The theoretical basis for the instrument is that life gains its quality from the ability and capacity of the individual to satisfy his or her needs. The scale covers emotional reactions, social isolation, energy level, sleep, physical mobility, and pain. The QLDS, consists of 34 items related to depression, scored binomially (0-1); numerically higher scores depict lower QOL.

### The Multidimensional Scale of Perceived Social Support [23]

The MSPSS is a 12-item questionnaire that assesses participants' perception of the role that friends, family and peers play in their lives. Respondents rate items on a seven-point Likert scale with higher scores corresponding to greater social support. The total social support score was used in this study.

### The Rating of Perceived Exertion Scale [15]

The RPE is a standard assessment of physical activity intensity. Perceived exertion is how hard you feel your body is working. It is based on the physical sensations a person experiences during physical activity, including increased heart rate, increased respiration or breathing rate, increased sweating, and muscle fatigue. The scale ranges from 6 to 20, where 6 is "no exertion at all" and 20 "maximal exertion.

Heart rate was measured by chest monitors and we recorded the number of exercise sessions each participant completed.

## Results

Table 2 shows the baseline characteristics of the intervention and active comparator groups.

**Table 2:** Pragmatic RCT of an exercise programme for women with depression: baseline characteristics of study groups

	Intervention arm (*n *= 19)	Active comparator arm (*n *= 19)
Mean age (years) (SD)	57.0 (9.9)	50.4 (15.2)
Marital status, *n *(%)		
Single	9 (47.4)	9 (47.4)
Married	9 (47.4)	10 (52.6)
Widowed	1 (5.3)	0 (0)
Psychiatric medication, *n *(%)		
Yes	15 (78.9)	17 (89.5)
No	4 (21.1)	2 (10.5)
Talking therapy, *n *(%)		
Yes	6 (31.6)	11 (57.9)
No	13 (68.4)	8 (42.1)
Mean BDI score (SD)	26.5 (10.7)	30.5 (12.0)
Mean GHQ score (SD)	8.3 (3.7)	8.8 (3.4)
Mean SF-12 score (SD)	25.9 (6.2)	25.6 (7.5)
Mean RSES score (SD)	11.3 (5.8)	11.0 (4.9)
Mean MSPSS score (SD)	47.6 (18.3)	51.6 (15.4)
Mean QLDS score (SD)	15.5 (7.9)	20.3 (8.5)
Mean heart rate @ time 0 (beats/min) (SD)	87.5 (15.8)	92.8 (5.6)
Mean peak heart rate (beats/min) (SD)	109.6 (21.1)	121.3 (15.8)
Mean RPE score @ time 0 (SD)	9.2 (3.2)	8.6 (3.3)

As shown in table 2 both groups were similar at baseline in terms of demographic characteristics and scores on the study outcome measures.

Table 1 shows the outcomes between the two groups of the study.

Compared with the active comparator group, the preferred intensity group showed significantly lower depression levels, higher self-esteem levels, better general mental health, and improved quality of life. General health and perceived social support improved for both groups, but there was no statistically significant difference between them. Heart rate and perceived effort increased for both groups, but these differences were not statistically significant. However, at peak exertion, whilst the intervention group experienced less effort by the end of the exercise sessions, the active comparator group experienced significantly more exertion. The intervention group attended a greater number of exercise sessions (8 (66%) out of 12 sessions) than the active comparator group (6 (50%) out of 12 sessions), giving a mean difference of 2.3 sessions (95% CI -0.3 to 4.8). The effect size was medium (*d *= 0.58). An independent *t*-test showed that the difference between the groups was significant (t = 1.781, df = 36, p = 0.0415, one-tailed).

## Discussion

### Exercise and Depression

We have shown that exercise of preferred intensity improves depressive symptoms, general health and well-being and exercise adherence rates and this finding confirms the positive results shown by earlier studies of clinical populations [8]. Calculating effect size for the primary outcome using Cohen's method and a standardised mean difference (SMD) to draw comparisons with the 2010 Cochrane review [8], the effect size of 0.71 is larger than the moderate, non-significant SMD reported previously; exercise of preferred intensity appears to promote greater benefits.

### Intensity and depression outcomes

To our best knowledge, no previous trials tested the effect of preferred intensity exercise. Our focus on preferred intensity has generated results that confirm the effects of exercise in reducing depression whether assessed by symptom severity or by measures such as BDI scores [8]. The positive effects of exercise on the range of psychological (BDI, GHQ-12, RSES scores), social (QLDS and MDSPSS scores) and physiological (RPE and Heart Rate scores) outcomes as shown here have not been studied previously. A concomitant qualitative study conducted as an adjunct to this trial has shown the value of preferred intensity towards the achievement of improved outcomes [24] as it introduced the exercise gently and incrementally, gave the participants control over the levels of intensity they could handle and appeared to increase their enjoyment of the exercise. Until now, little has been known about the effect of preferred intensity exercise. National guidelines continue to recommend frequency and intensity levels shown to be overly ambitious among middle-aged women with depression [24]. We have shown that exercise of nationally recommended frequency for depressed women and preferred intensity generated improvements across a range of health, well being and social outcomes.

### Adherence rates

Adherence rates range from 59% to 95%, an average of 82% [8], but few were clinical populations of women. Adherence rates for Middle-aged depressed women are not well known, previous qualitative research reports low levels of attendance in this group [25]. Our attendance rates of 66% for the intervention are impressive in light of the paucity of previous research.

### The addition of psycho-educational interventions

The addition of group aerobic exercise sessions, guided by a qualified exercise therapist with manualised psycho-educational interventions is seldom found in studies of clinical populations. In the UK, exercise has been introduced into services as a treatment for depression under exercise on prescription schemes, whereby patients receive vouchers from their GP, for example, to use at a local gym. A recent systematic review and comparative trial of these schemes has shown them to have limited effectiveness [26,27]. We hypothesised that to generate the improvements across a range of outcomes among depressed women who are largely sedentary; exercise must be accompanied by supportive psychosocial interventions. This hypothesis has been confirmed by our findings.

## Strengths

This work impacts the science in this are in several ways. We have tested a preferred versus prescribed intensity exercise intervention with concomitant manualised psychosocial support, in a clinical population under-represented in previous studies against a range of mental and general health and well being outcomes, not captured in previous studies that have improved substantially as a result of the interventions we introduced. The exercise adherence rates are impressive in light of previous findings among healthy and help-seeking populations that show relatively poor adherence.

## Limitations

The loss of participants once enrolled is common in exercise trials involving clinical populations, but is a limitation in this study, necessitated in part, by our attempts to minimise the inconvenience to our participants by localising where the programmes occurred. We were unable to follow up the participants formally due to funding constraints and this is a potential limitation. Finally, assessors of the outcome measures were not blinded to the allocation of participants to each arm of the trial. It is our view that this did not have a negative impact on findings, but it is a potential limitation.

## Conclusions

Preferred intensity exercise coupled with motivational education and support is likely to improve health and quality of life of women living with depression and improve their exercise adherence rates. The key to the improvements shown here is 'mentored' exercise, which included group motivational support and a low effort walking plan. Exercise tailored to preferred exertion levels, combined with support from others is a prescription designed to improve depressed women's overall health and well being.

## Competing interests

The authors declare that they have no competing interests.

## Authors' informatiom

Patrick Callaghan is a Professor of Mental Health Nursing at the Faculty of Medicine and Health Sciences, University of Nottingham.

Elizabeth Khalil is a Research Fellow at the Faculty of Medicine and Health Sciences, University of Nottingham.

Ioannis Morres was formerly a Research Associate at the Faculty of Medicine and Health Sciences, University of Nottingham.

Tim Carter is a PhD Student at the Faculty of Medicine and Health Sciences, University of Nottingham.

## Authors' contributions

PC, LK conceived of the study and participated in its design and coordination, and helped to draft the manuscript. IM conceived of the study and participated in its design and coordination. LK and IM ran the exercise interventions. TC helped to draft the manuscript. All authors read and approved the manuscript.

## Pre-publication history

The pre-publication history for this paper can be accessed here:

http://www.biomedcentral.com/1471-2458/11/465/prepub
